# Attenuated and normalized item-item product network for sequential recommendation

**DOI:** 10.7717/peerj-cs.867

**Published:** 2022-01-21

**Authors:** Weiqiang Di, Zhihao Wu, Youfang Lin

**Affiliations:** School of Computer and Information Technology, Beijing Jiaotong University, Beijing, China

**Keywords:** Sequential recommendation, Recommendation, Item co-occurrence, Item-item product

## Abstract

Sequential recommendation has become a research trending that exploits user’s recent behaviors for recommendation. The user-item interactions contain a sequential dependency that we need to capture to better recommend. Item-item Product (IIP), which models item co-occurrence, has shown good potential by characterizing the pairwise item relationships. Generally, recent behaviors have a greater impact on the current than long-term historical behaviors. And the decaying rate of influence around infrequent behaviors is fast. However, IIP ignores such a phenomenon when considering item-item relevance and leads to suboptimal performance. In this paper, we propose an attenuated IIP mechanism which is position-aware and decays the influence of historical items at an exponential rate. Besides, In order to make up for scenarios where the influence is not in a monotonous decline trend, we add another normalized IIP mechanism to complement the attenuated IIP mechanism. It also strengthen the model’s ability in discriminating favorite items under the sparse data condition by enlarging the gap of matching degree between items. Experiments conducted on five real-world datasets demonstrate that our proposed model achieves better performance than a set of state-of-the-art sequential recommendation models.

## Introduction

The recommendation system can help to alleviate the problem of information overload by collecting information, analyzing interests and then selecting items we might be interested in proactively from lots of possible options. It has become increasingly important in today’s information-rich era. Different from traditional collaborative filtering, which has the assumption that users who have similar interactions historically will like to have similar tastes in the future, sequential recommendation has attracted lots of attention recently distinguished by paying more attention to user’ interaction context. A user’s behavior usually exhibits some sequential dependency. More characterization of the interaction context can take such dependency into account and lead to more accurate recommendation.

**Figure 1:**
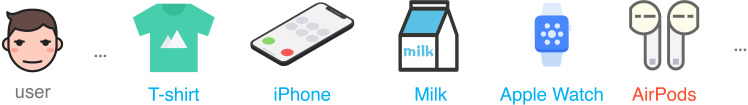
An example of sequential recommender systems with online shopping.

In sequential recommendation, we predict the next item that may be of interest based on users’ recent interactions. There are two keys that can profoundly affect the accuracy of sequential recommendation which we need to focus on: (i) capturing user’s short-term interests, and (ii) learning user’s long-term preference. In the short-term part, it is of great importance to capture the sequential dependency existing in the sequence between the target item and items that user has recently interacted. For instance, as shown in Fig. 1, the purchase of an iPhone in history means a higher possibility of buying AirPods than buying a T-shirt. The purchase of both iPhone and Apple Watch further increase the likelihood of buying AirPods.

Methods considering different aspects have been proposed by far. For many recommender systems, a de facto solution is often based on Collaborative Filtering (CF) techniques, as exemplified by Matrix Factorization (MF) and Neural network-based Collaborative Filtering algorithms (Koren, Bell & Volinsky, 2009; He et al., 2017). Increasing interests have been put in sequential recommendation recently. For modeling sequential patterns, models using Recurrent Neural Networks (RNN) are proposed which emphasize the importance of temporal dependency (Hidasi et al., 2015; Wang et al., 2015). While effective, they have the shortcoming of poor parallelization and still struggle at capturing long-term dependency. Convolutional Neural Networks (CNN) have been introduced for the excellent ability in capturing local dependency and parallelization. One of the outstanding representative Caser (Tang & Wang, 2018) applied CNN in sequential recommendation task and achieved superior performance. The self-attention mechanism (Vaswani et al., 2017) is very successful in natural language processing and has been well applied in this field with SASRec (Kang & McAuley, 2018) for example. It has the advantage in capturing long-term dependency. MA-GNN (Ma et al., 2020) makes a pioneering work in exploring the potential of graph structure and has got a good performance. HGN (Ma, Kang & Liu, 2019) models item co-occurrence in the Item-item Product(IIP) module, which is computationally efficient and has excellent performance in sequential recommendation. Successful learning of pairwise item relationships (PIR) can play a significant role in recommender systems (Ning, Desrosiers & Karypis, 2015). The effectiveness of such pattern has been verified in papers of item-based CF such as NARM (Li et al., 2017), NAIS (He et al., 2018) and NPE (Nguyen & Takasu, 2018). Compared to models using RNN, PIR-based models have stronger capabilities in capturing the item co-occurrence patterns. As for CNN and self-attention models, they require much more parameters to learn and can easily overfit under sparse datasets like those used in our paper. Compared with self-attention models, CNN models are less flexible in modeling relations between any two items. Despite its success, IIP ignores dynamics of influence when considering the matching degree between historical items and the target item. In practice, a common phenomenon is that the influence of past behaviors on current behavior becomes smaller as time goes by and the rate of decaying is getting faster. Failure to consider such a characteristic prevents IIP from achieving better performance.

To solve the problem mentioned above, we propose an attenuated and normalized item-item product network (ANIIP) for sequential recommendation. It is position-aware which strengthens IIP by taking the decaying effect of influence into account. Specifically, we attenuate the weight of matching degree between each historical item and the target item exponentially as the position moves away from the current time step. User-item interactions sometimes have complex dependency relationship which does not apply to the decaying influence scenario mentioned above. Besides, in datasets where user behaviors are sparse, model’s ability to distinguish items that users prefer is insufficient. The score for item of interest cannot form a sufficient gap from items not of interest to users. Considering such situation, we add another normalized IIP dealing with more dynamical dependencies to compensate that.

In summary, the contributions of this work are as follows:

• We propose an attenuated IIP mechanism which considers the diminishing effect of item’s influence. In attenuated IIP, it is position-aware where the weight of influence in each historical position is attenuated exponentially as the position moves away from the current time step.
• We complement a normalized IIP mechanism to consider situations where the decaying influence scenario dose not apply, which also employs exponential function to enlarge the difference between the similarity scores so as to enhance the model’s discrimination ability in sparse datasets.
• We conducted thorough experiments to evaluate our model on five real-world datasets and show the effectiveness of our method over the state-of-the-art baselines for sequential recommendation.

## Related Work

The sequential recommendation task is different from the general recommendation task mentioned before. It aims to capture sequential patterns from items user has interacted with in order to predict next items accurately. A Markov chain is a classical way to solve this problem, which believes that the next behavior depends on previous behavior (Shani, Heckerman & Brafman, 2005). Then two notable methods are proposed: factorized personalized Markov chains (FPMC) (Rendle, Freudenthaler & Schmidt-Thieme, 2010) and Fossil (He & McAuley, 2016a). FPMC combines matrix factorization with Markov chains by embedding the transition information between adjacent interactions into the item latent factors to capture long-term and short-term interests of user. Fossil fuses similarity-based methods with Markov chains to make personalized sequential recommendation. With the development of deep neural networks in various fields, many methods apply RNN and CNN to encode past recorded sequence (Lai et al., 2018; Yuan et al., 2019). As the good ability in capturing the sequential patterns in sequence, RNN based methods (Hidasi et al., 2015; Du et al., 2016; Li et al., 2017; Hidasi & Karatzoglou, 2018) are widely used for the session-based recommendation task. Due to the strong local feature extraction capabilities, many methods of applying CNN have also been proposed. For instance, Caser (Tang & Wang, 2018) is a CNN based method with horizontal and vertical convolutional layer to process sequential items embedding matrix. On the other hand, self-attention mechanism (Zhang et al., 2018) is also utilized to infer the item-item relationship from user’s historical interactions. A Gating mechanism is adopted by HGN (Ma, Kang & Liu, 2019), a hierarchical gating network to select what item features can be passed to the downstream layers from the feature and instance levels. With the rapid development of graph neural networks in these two years, work on this line is also applied in sequential recommendation task due to their abilities to reveal the complex relationship between the item and the corresponding context in the sequence (Ma et al., 2020).

## Methodology

### Problem formulation

The recommendation task in our paper can be formulated as follows. Suppose there are a set of users *U* and a set of items *I*. Each user has a sequence of items he or she implicitly interacted with in the chronological order, denoted by ${S}^{u}=({S}_{1}^{u},{S}_{2}^{u},\ldots ,{S}_{ \left\vert {S}^{u} \right\vert }^{u})$, ${S}_{i}^{u}\in I$. To model the sequential recommendation task, for each user, we use the past subsequence of length *L* successive items ${S}_{1:L}^{u}(L\lt {|}{S}^{u}{|})$ as input and their next *T* items as the targets to be predicted.

### Overall architecture

**Figure 2:**
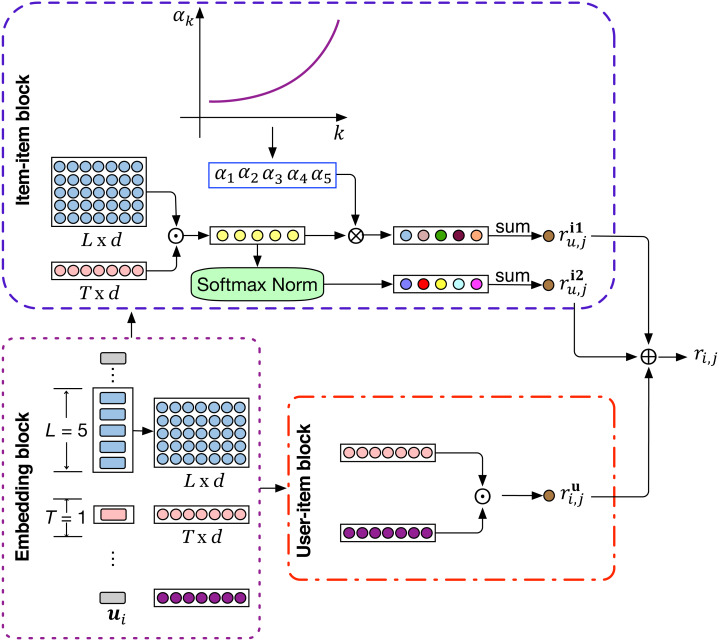
The model architecture of ANIIP. ⊙ The symbol denotes inner product, ⊕ denotes element-wise addition and ⊗ denotes element-wise multiplication. *T* is 1 in this figure for simplicity and 3 in practice.

In this section, we introduce the proposed Attenuated and Normalized Item-item Product network (ANIIP). It takes the decaying effect of influence into account when characterizing the matching degree between historical items and the target item. Figure 2 shows the overall architecture of ANIIP. First, for each user, we get the previous *L* items’ embeddings and the user embedding via the embedding layer. The embeddings obtained will then be processed by two different functional unit, namely user-item block and item-item block, to capture the general and the short-term interests separately. The user-item block characterizes the match degree between the user and the target item using the traditional matrix factorization method. The item-item block captures the relation between the target item and the historical items. Then, we combine the results from these two blocks as the final score of the item to be predicted.

### Embedding layer

As shown in Fig. 2, the input of our model is the user and accompanying sequence of *L* historical items, where the user and items are originally represented by a series of unique indexes. We convert each user and item into low-dimensional hidden features through the traditional look-up operation with the learnable user and item embedding table. We denote the user embedding matrix as ${\mathbi{E}}_{U}\in {\mathbb{R}}^{ \left\vert U \right\vert \times d}$, input item embedding matrix as ${\mathbi{E}}_{I}\in {\mathbb{R}}^{ \left\vert I \right\vert \times d}$ respectivly, where *d* is the size of latent dimension. Naturally, the sequence of previous *L* items of user *u* at time step *s* can be represented as ***E***_*u*,*s*_ ∈ ℝ^*L*×*d*^: And ***p***_*u*_ ∈ ℝ^*d*^ is the representation of user *u* in latent space.

### Attenuated IIP block

In sequential recommendation, recent interactive behaviors are very instructive for predicting the next item of interest. The reason is that sequential dependency usually exists in user-item interactions where some previous items influence the next item to be interacted. Among the many sequential dependency capturing methods, characterization of pairwise item relation is an important factor to capture which models patterns of item co-occurrence and be denoted as Item-item Product (IIP) in HGN (Ma, Kang & Liu, 2019). In IIP, we compute the matching degree through inner product between the embeddings of target item and recent interacted historical items:

(1) \begin{eqnarray*}{\hat {r}}_{u,j}^{i}=\sum _{{\mathbi{E}}_{k}\in {\mathbi{E}}_{u,s}}{\mathbi{q}}_{j}\cdot {\mathbi{E}}_{k}\end{eqnarray*}

where ***q***_*j*_ ∈ ℝ^*d*×1^ is the output embedding of the target item. Note that an item has two embedding representations to distinguish its role of as the prediction item or as a historical item. IIP adds the matching degree between each historical item and the target item to synthesize their influence.

Compared with many recent models (Tang & Wang, 2018; Kang & McAuley, 2018), it is very effective and has more interpretability (Deshpande & Karypis, 2004; Ma, Kang & Liu, 2019). It is more suitable to capture co-occurrence patterns of items. This is important since prediction relies on collective dependencies in sequential recommendation and co-occurrence patterns present this dependency very concisely and powerfully.

Although IIP performs well, it does not consider the decaying effect of historical items’ influence on the matching degree. This is obviously contrary to our common sense. Usually, recent behaviors have more influence on the next action than behaviors happened long time ago. For example, given a user that has purchased an iPad, the user is much more likely to buy an iPad protector in the short term. But the strong correlation will decline rapidly over time with some possible reasons. It may be that the user has already obtained it from other platforms or he/her doesn’t like to put on a protector on the iPad.

Under the guidance of this idea, we adjust the influence of historical items at different locations. The farther the item is from the current time step, the lower its correlation with the target item, and decline at an exponential rate. The formula of the attenuated IIP is thus as follows:

(2) \begin{eqnarray*}\begin{array}{@{}r@{}} \displaystyle {\alpha }_{k} & ={m}^{k} & \\ \displaystyle {\hat {r}}_{u,j}^{i1} & =\sum _{{\mathbi{E}}_{k}\in {\mathbi{E}}_{u,s}}{\alpha }_{k}({\mathbi{q}}_{j}\cdot {\mathbi{E}}_{k}) \end{array}\end{eqnarray*}

where *α*_*k*_ are the attenuation coefficients applied on the matching degrees when computing IIP, *m* is the exponential decay constant (*e.g.*, 0.8) that determines the decaying rate as the distance increases with respect to the current time step and *k* is the span of time steps between the current to the historical position, which from near to far can be 1, 2, 3, 4,…,etc. ***q***_*j*_ is the output embedding of the target item.

### Normalized IIP block

Sometimes, historical items far away from the target item may have great impact which cannot be coped well with attenuated IIP. We thus preserve an un-attenuated IIP block (*e.g.*, IIP block) considering that. Furthermore, trained model in sparse datasets has a problem in distinguishing items that users prefer due to the scarcity of user preferences. This leads to the insufficient gap of matching scores between preferred items and items not of interest to users. To improve that, we exploit the exponential function to enlarge such gap between scores in IIP.

(3) \begin{eqnarray*}\begin{array}{@{}r@{}} \displaystyle {\beta }_{k} & = \frac{\exp \nolimits \left( {\mathbi{q}}_{j}\cdot {\mathbi{E}}_{k} \right) }{\sum _{k=1}^{L}\exp \nolimits \left( {\mathbi{q}}_{j}\cdot {\mathbi{E}}_{k} \right) } . & \\ \displaystyle {\hat {r}}_{u,j}^{i2} & =\sum _{{\mathbi{E}}_{k}\in {\mathbi{E}}_{u,s}}{\beta }_{k}({\mathbi{q}}_{j}\cdot {\mathbi{E}}_{k}) \end{array}\end{eqnarray*}

### Prediction layer

Previously, we have introduced the core modules studying how to impose influence dynamics when modeling item co-occurrence characteristics under sparse datasets. Besides, the user’s long-term preference is also an important factor for recommendation. We consider that in our User-item block as shown in the model architecture diagram and adopt the classical matrix factorization to capture that and denote it as ${\hat {r}}_{u,j}^{u}={\mathbi{p}}_{u}^{\mathrm{T}}{\mathbi{q}}_{j}$ where ***p***_*u*_ is the user embedding and ***q***_*j*_ is the target item. The final score integrates the long-term and short-term interests of the user to produce more accurate prediction. Given user *u* and a sequence of *L* recent items ***E***_*u*,*s*_ at time step *s*, the prediction score of user *u* on item *j* is:

(4) \begin{eqnarray*}\begin{array}{@{}r@{}} \displaystyle {\hat {r}}_{u,j}={\hat {r}}_{u,j}^{u}+{\gamma }_{1}\ast {\hat {r}}_{u,j}^{i1}+{\gamma }_{2}\ast {\hat {r}}_{u,j}^{i2} \end{array}\end{eqnarray*}

where *γ*_1_ and *γ*_2_ are two trainable parameter to adjust their proportions in different scenarios. In order to limit the two coefficients, they are passed to a sigmoid function before applying to ${\hat {r}}_{u,j}^{i1}$ and ${\hat {r}}_{u,j}^{i2}$.

### Network training

In this paper, we utilize Bayesian Personalized Ranking (BPR) objective to optimize the proposed model in training. BPR is a classic pairwise ranking method, which aims to rank the observed next item (positive) ahead of the accompanying negative samples. We select BPR based on our data content. The processed data records users’ implicit behavior, which means only positive interactions are available. The items that user has not been recorded yet can still be divided into two categories: items the user does not like and items with no interaction at the moment but may be interested in the future. It is obviously inappropriate to optimize the labels of these potentially interesting items as negative samples. The formula of BPR is as follows:

\begin{eqnarray*}{\mathrm{arg~ min}}_{\Theta }\sum _{u,{L}_{u}^{s},j,{j}^{{^{\prime}}}}-log\sigma ({\hat {r}}_{u,j}-{\hat {r}}_{u,{j}^{{^{\prime}}}})+\lambda (\parallel \Theta {\parallel }^{2}) \end{eqnarray*}

where ${L}_{u}^{s}$ denotes *L* successive items of user *u* at time step *s*, *j* denotes the positive items user *u* will interact with at time step *s*, and we randomly sample one negative instance for each target item *j*, denoted by *j*′.Θ denotes all the trainable parameters in our model. *λ* is the coefficient for regularization. In addition, we pretrain our model by initializing the user embeddings and item embeddings with the trained embeddings in HGN (Ma, Kang & Liu, 2019). This can speed up the rate of convergence and help achieve better performance which we will show in the part of ablation study.

## Evaluation

In this section, we first introduce the datasets, evaluation metric, baseline methods and experimental settings. And then we report the experimental results and analyze the effectiveness of the proposed model.

### Datasets

To fully evaluate the capability of our model, we do experiments on five real-world datasets.^1^ 1The data is available on GitHub, released by the authors of HGN: https://github.com/allenjack/HGN.

• *MovieLens-20M* (Harper & Konstan, 2015). User-Movie dataset is one of the popular benchmark datasets collected from the MovieLens website. It totally has 20 million user-movie interactions.
• *Amazon-Books* (He & McAuley, 2016b). Amazon is a well-known e-commerce platform. The dataset is collected from the Amazon review dataset with category Books.
• *Amazon-CDs* (He & McAuley, 2016b). The dataset is also adopted from the Amazon review dataset, but with category CDs.
• *Goodreads-Comics* (Kang & McAuley, 2018) The dataset mainly contains the data of the genres of Children from the goodreads website.
• *Goodreads-Children* (Kang & McAuley, 2018). Goodreads is a social network for reading-sharing. The dataset is collected in 2017 from the goodreads website with a focus on the genres of Children.

We preprocess datasets before the experiments. Following previous methods using the same datasets (Ma, Kang & Liu, 2019), we convert rating data to implicit feedback data for that in real-world, most feedback is not explicit but implicit. To ensure the quality of data, we filter out users with less than 10 ratings and items appearing less than 5 times. Table 1 summarizes the statistics of them.

**Table 1:** The statistics of the datasets.

Dataset	#Users	#Items	#Interactions	Density
*CDs*	17,052	35,118	472,265	0.079%
*Books*	52,406	41,264	1,856,747	0.086%
*Comics*	34,445	33,121	2,411,314	0.211%
*Children*	48,296	32,871	2,784,423	0.175%
*ML20M*	129,797	13,649	9,921,393	0.560%

As for the division of datasets, for each user, we split the interactions into training, validation and test sets in chronological order and the proportion is 70%, 10% and 20% respectively. Each model is executed five times and the average score are reported.

### Evaluation metrics

We evaluate all the methods by Recall@ *K* and NDCG@ *K*. For each user, given a list of top *K* predicted items of the user by each method, and the items in his/her test set. Recall@ *K* indicates what percentage of items in user test set emerge in the top *K* recommended list. NDCG@ *K* is the normalized discounted cumulative gain at *K*, which takes the position of correctly recommended items into account. *K* is set to 10.

### Comparison methods

We compare our model wih the following baselines:

• **BPRMF**. A classic method applying Bayesian Personalized Ranking to Matrix Factorization (Rendle et al., 2009) for recommendation.
• **GRU4Rec**. A RNN-based approach to model item sequences (Hidasi et al., 2015) for session-based recommendations.
• **GRU4Rec+**. An improved version of GRU4Rec (Hidasi & Karatzoglou, 2018), which adopts an advanced loss function and sampling strategy.
• **Caser**. A CNN-based to capture both general preferences and sequential patterns, and capture skip behaviors (Tang & Wang, 2018) for recommendation.
• **SASRec**. Applying self-attention mechanism (Kang & McAuley, 2018) to infer item-item relationship for prediction.
• **GC-SAN**. A method which performs session-based recommendation by using both graph neural network and self-attention mechanism (Xu et al., 2019).
• **MARank**. An approach unifying individual- and union-level item interactions to infer user preference from multiple views (Yu et al., 2019).
• **HGN**. A hierarchical gating network with feature gating and instance gating to select effective information, and item-item product module to capture item relations (Ma, Kang & Liu, 2019) for sequence recommendation.
• **MA-GNN**. A method combining memory network and graph neural network for capturing the short-term and long-term interests of users, simultaneously adopting a bilinear function rather than inner product to capture the item-item relations (Ma et al., 2020).

### Implementation details

Following the same setting in MA-GNN and HGN, we set |*L*| = 5 and |*T*| = 3. The embedding size of our model is set to 50 for fair comparison. Adam optimizer is used with 10^−3^ as the initial learning rate. L2 regularization is also used with weight decay set to 10^−3^ and the batch size of our model is 1024. The exponential decay constant *m* is selected from {0.1, 0.2, 0.3, 0.4, 0.5, 0.6, 0.7, 0.8, 0.9}. We adopt an early stop strategy which stops training when the validation metric does not have improvements for 5 consecutive epochs. As for the historical length, because the long sequence does not have much gain for the model, we are consistent with the HGN without change. In order to make the results stable, we use the average of 5 runs. The codes are implemented using PyTorch. For baseline methods, since this research is based on the source code and data of HGN, and the results of HGN and some other models running on our machine are basically consistent with the results in HGN, we thus use the results reported in HGN.

**Table 2:** The performance of all methods.

	CDs		Books		Comics		Children		ML20M	
	R@10	N@10	R@10	N@10	R@10	N@10	R@10	N@10	R@10	N@10
BPRMF	0.0269	0.0145	0.0260	0.0151	0.0788	0.0713	0.0814	0.0664	0.0774	0.0785
GRU4Rec	0.0302	0.0154	0.0266	0.0157	0.0958	0.0912	0.0857	0.0715	0.0804	0.0912
GRU4Rec+	0.0356	0.0171	0.0301	0.0171	0.1288	0.1328	0.0978	0.0821	0.0904	0.0946
Caser	0.0297	0.0163	0.0297	0.0216	0.1473	0.1529	0.1060	0.0943	0.1169	0.1116
SASRec	0.0341	0.0193	0.0358	0.0240	0.1494	0.1592	0.1165	0.1007	0.1069	0.1014
GC-SAN	0.0372	0.0196	0.0344	0.0256	0.1490	0.1563	0.1099	0.0967	0.1161	0.1119
MARank	0.0382	0.0151	0.0355	0.0223	0.1325	0.1431	0.1092	0.0980	0.1002	0.1031
HGN	0.0426	0.0233	0.0429	0.0298	0.1743	0.1927	0.1263	0.1130	0.1255	0.1195
MA-GNN	0.0442	0.0214	0.0432	0.0279	0.1617	0.1655	0.1215	0.1137	0.1236	0.1272
ANIIP	**0.0554**	**0.0481**	**0.0529**	**0.0534**	**0.1976**	**0.2578**	**0.1410**	**0.1571**	**0.1310**	**0.1530**

**Note.**

Best performing method is shown in bold. The second best performing method is shown with an underline.

### Experimental results

Let’s compare the performance of our model and the baselines first. Table 2 summarizes the overall results on five datasets. Obviously our model performs best among them.

We can first note that all sequential models behave better than the non-sequential BPRMF. The reason can be due to the dynamic sequential dependencies between the recent interactions and the target item. This clearly shows the significance of user’s recent interactions for prediction. We also find that the performance of CNN-based model Caser is better than RNN-based models like GRU4Rec and GRU4Rec+. It shows the strong potential of convolutions in sequential recommendation. This may be attributed to that convolutions allow a more flexible order over user-item interactions and do better at capturing collective dependencies which is not available in RNN.

We can also observe that SASRec and GC-SAN, which uses self-attention, behaves better than Caser most of the time. This shows the advantage of using self-attention in learning sequential dependency. MA-GNN and HGN perform very well compared to other baselines. This can be due to their effective mechanism for solving both long-term and short-term interests and the modeling of item co-occurrence. Besides, graph-convolution based MA-GNN performs worse than HGN, which indicates that the potential of graph convolution in sequential recommendation needs to be further explored.

We note that our model consistently outperforms the baselines on all metrics and all datasets with a large margin, which clearly demonstrate the effectiveness of the model design. The excellent performance of ANIIP illustrates that capturing item co-occurrence patterns is important and IIP is a very effective way when position-aware attenuated influence and normalized influence are both considered and fused. We will show in the ablation study the complementarity of attenuated IIP block and normalized IIP block.

The time complexity of our model is also very low. The key part of our algorithm is to calculate the attenuation coefficient and the normalization coefficient on the matching degree between the target item and the historical items. The calculation are scaled to the length of history, that is, the algorithm has a linear complexity *O*(*dL*), where *d* is the dimension of embedding and *L* is the length of history. The time complexity of CNN models are *O*(*d*^2^*k*), where *k* is the kernel width. The time complexity of self-attention models are *O*(*L*^2^*d*). Among the models that consider historical items, it is in the category of the least complex. Besides, the improvements in NDCG are much larger than those in Recall, which shows that our model can not only identify the items users like, but also rank them high.

### Ablation analysis

To show the contribution of different model designs, we conduct an ablation study here and summarize the results in Tables 3 and 4 for Recall@10 and NDCG@10 separately. We denote our model having only the attenuated IIP block and the MF part as IIP-A, which is to remove the term ${\hat {r}}_{u,j}^{i2}$ in Eq. (4). The model having only the normalized IIP block and the MF part is denoted as IIP-N, which is to remove the term ${\hat {r}}_{u,j}^{i2}$ in Eq. (4). We denote IIP as replacing the ${\hat {r}}_{u,j}^{i1}$ and ${\hat {r}}_{u,j}^{i2}$ part in Eq. (4) with ${\hat {r}}_{u,j}^{i}$ in Eq. (1) if expressed in formula.

**Table 3:** The ablation analysis (Recall@10) on five datasets.

Datasets	CDs	Books	Comics	Children	ML20M
IIP	0.0489	0.0450	0.1751	0.1265	0.1272
IIP-N	0.0522	0.0471	0.1844	0.1299	0.1114
IIP-A	0.0527	0.0482	0.1895	0.1399	0.1311
ANIIP w/o norm	0.0490	0.0465	0.1829	0.1313	0.1275
ANIIP w/o pretrain	0.0532	0.0518	0.1952	**0.1414**	**0.1314**
ANIIP	**0.0554**	**0.0529**	**0.1976**	0.1410	0.1310

**Table 4:** The ablation analysis (NDCG@10) on five datasets.

Datasets	CDs	Books	Comics	Children	ML20M
IIP	0.0423	0.0453	0.2276	0.1401	0.1481
IIP-N	0.0441	0.0459	0.2335	0.1427	0.1313
IIP-A	0.0456	0.0479	0.2440	0.1555	0.1529
ANIIP w/o norm	0.0424	0.0466	0.2384	0.1453	0.1480
ANIIP w/o pretrain	0.0458	0.0521	0.2533	0.1570	**0.1534**
ANIIP	**0.0481**	**0.0534**	**0.2578**	**0.1571**	0.1530

Let’s first take a look at the effect of our core attenuation mechanism. We can see from the results that the performance of our attenuated IIP (*e.g.*, IIP-A) has been greatly improved compared to the pure IIP (*e.g.*, IIP). This fully demonstrates the correctness of our idea about attenuated influence of historical items in IIP when they are farther away from the current time step. Similar improvements can also be noted in IIP-N. Through the exponential function, we enlarge the difference between the matching scores in IIP, thereby to improve the model’s ability of discrimination . The positive effect of this mechanism decreases as the data sparsity decreases. This is because richer behavioral data can train the model parameters better and improve the discrimination ability of the model. To further demonstrate the role of normalization in the normalized IIP block. We remove the normalization step and just keep the ordinary IIP in it. This variant is denoted as ANIIP w/o norm. From the results, we can see that combining the attenuated IIP block and the IIP block is even worse than the performance of IIP-A. This proves the necessity of our normalization applied in the IIP block. We can note that ANIIP outperforms both IIP-A and IIP-N, this shows that the attenuated IIP block and normalized IIP block are complementary. Besides, the attenuated IIP mechanism is more important than normalized IIP mechanism from the declining performance removing them individually.

Our model uses pre-training. We apply the user embeddings and item embeddings trained by the HGN model (Ma, Kang & Liu, 2019) to initialize our model parameters. The effect removing pre-training is recorded in the row named ANIIP w/o pretrain. We can see that pre-training is more helpful for sparser datasets like CDs and Books. It is difficult to learn good patterns on sparse datasets. At this time, exploiting patterns mined by other models is meaningful.

**Figure 3:**
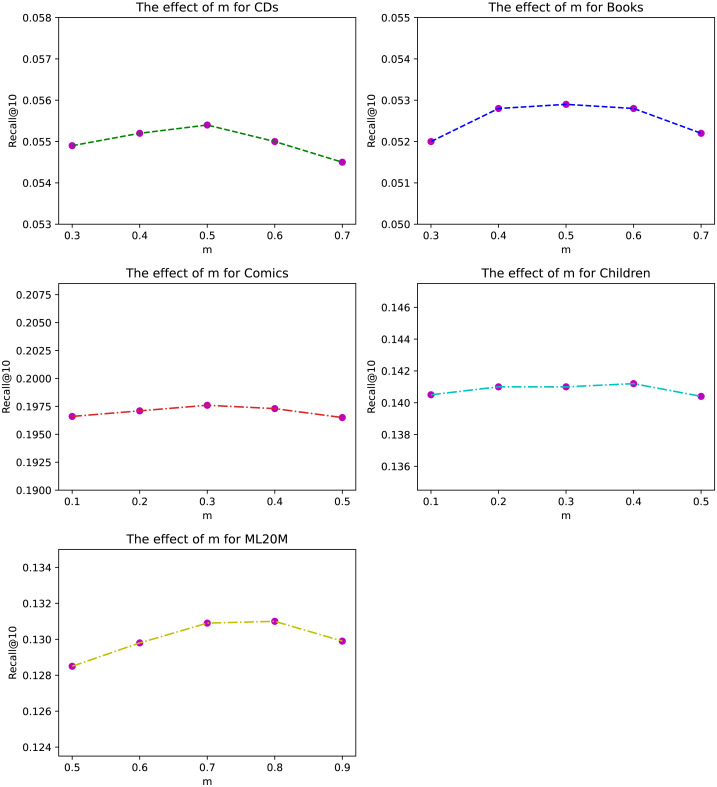
The effect of exponential decay constant *m* for different datasets under Recall@10.

### Influence of hyper-parameters

The exponential decay constant *m* of ANIIP is an important hyper-parameter in our proposed model. We investigate its effects in Fig. 3 on our datasets. From the results, we can observe that within the appropriate value range in each dataset, the fluctuations are very small, which shows the stability of our method. Besides, compared to sparse datasets CDs and Books, the optimal *m* in Comics is smaller. This may be due to that in denser datasets, the items that are closer to the current time step are more meaningful. Thus the attenuation rate of their influence should be quicker. For ML20M, the optimal *m* is the largest, which shows that recent history items are all important for predicting the future.

**Figure 4:**
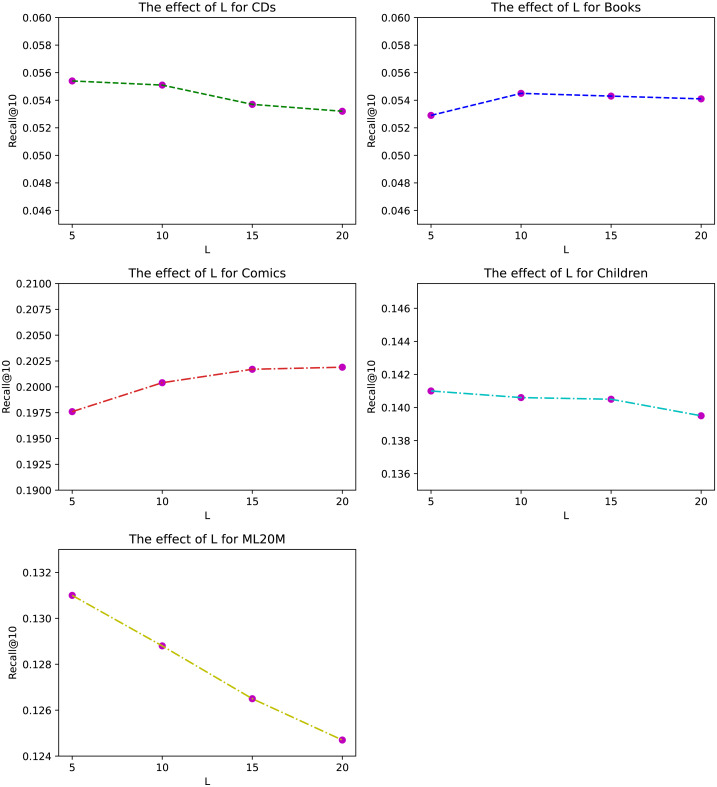
The effect of historical length *L* for different datasets under Recall@10.

The length of history *L* of ANIIP is also an hyper-parameter in our proposed model. We investigate its effects in Fig. 4. From the results, we can observe that the optimal length is not very large for most datasets except for Comics. This is because the frequency of people’s buying behavior is not very high, leading to the fact that the importance of the historical sequence far away from the current moment is a bit small. The significance of our model is that it considers the rapid decline of influence of historical items as the distance increases between the target item and the historical item. Combined with the normalized attention mechanism, ANIIP makes our recommendation ability to a new level.

## Conclusion

In this paper, we present ANIIP, which takes the dynamics of influence into consideration and combine it with the effective item co-occurrence modeling mechanism IIP. ANIIP decays the influence of historical items exponentially from the current time step. And at the same time complements a normalized IIP block to consider situations where the decaying influence scenario dose not apply. Experimental results on five real-world datasets demonstrate the effectiveness of our model.

## Supplemental Information

10.7717/peerj-cs.867/supp-1 Supplemental Information 1 The code and data for our model

## Additional Information and Declarations

### Data Availability

The following information was supplied regarding data availability: The raw measurements are available in the Supplementary Files. The data is available on GitHub released by the authors of HGN: https://github.com/allenjack/HGN.
